# Cerebral Pericytes and Endothelial Cells Communicate through Inflammasome-Dependent Signals

**DOI:** 10.3390/ijms22116122

**Published:** 2021-06-06

**Authors:** Mihály Kozma, Ádám Mészáros, Ádám Nyúl-Tóth, Kinga Molnár, Laura Costea, Zsófia Hernádi, Csilla Fazakas, Attila E. Farkas, Imola Wilhelm, István A. Krizbai

**Affiliations:** 1 Institute of Biophysics, Biological Research Centre, Eötvös Loránd Research Network (ELKH), 6726 Szeged, Hungary; kozma.mihaly@brc.hu (M.K.); meszaros.adam@brc.hu (Á.M.); nyul-toth.adam@brc.hu (Á.N.-T.); molnar.kinga@brc.hu (K.M.); zsofia.hernadi@gmail.com (Z.H.); fazakas.csilla@brc.hu (C.F.); farkas.attilae@brc.hu (A.E.F.); 2 Theoretical Medicine Doctoral School, University of Szeged, 6720 Szeged, Hungary; 3 Doctoral School of Biology, University of Szeged, 6726 Szeged, Hungary; 4 Vascular Cognitive Impairment and Neurodegeneration Program, Oklahoma Center for Geroscience and Healthy Brain Aging, Department of Biochemistry and Molecular Biology, University of Oklahoma Health Sciences Center, Oklahoma City, OK 73104, USA; 5 Department of Physiology, Anatomy and Neuroscience, Faculty of Science and Informatics, University of Szeged, 6726 Szeged, Hungary; 6 Institute of Life Sciences, Vasile Goldiş Western University of Arad, 310025 Arad, Romania; laura.m.costea@gmail.com; 7 Foundation for the Future of Biomedical Sciences in Szeged, Szeged Scientists Academy, 6720 Szeged, Hungary

**Keywords:** blood–brain barrier, brain pericyte, cerebral endothelial cell, interleukin-1β, inflammasome, neuroinflammation, neurovascular unit, tight junctions

## Abstract

By upregulation of cell adhesion molecules and secretion of proinflammatory cytokines, cells of the neurovascular unit, including pericytes and endothelial cells, actively participate in neuroinflammatory reactions. As previously shown, both cell types can activate inflammasomes, cerebral endothelial cells (CECs) through the canonical pathway, while pericytes only through the noncanonical pathway. Using complex in vitro models, we demonstrate here that the noncanonical inflammasome pathway can be induced in CECs as well, leading to a further increase in the secretion of active interleukin-1β over that observed in response to activation of the canonical pathway. In parallel, a more pronounced disruption of tight junctions takes place. We also show that CECs respond to inflammatory stimuli coming from both the apical/blood and the basolateral/brain directions. As a result, CECs can detect factors secreted by pericytes in which the noncanonical inflammasome pathway is activated and respond with inflammatory activation and impairment of the barrier properties. In addition, upon sensing inflammatory signals, CECs release inflammatory factors toward both the blood and the brain sides. Consequently, CECs activate pericytes by upregulating their expression of NLRP3 (NOD-, LRR-, and pyrin domain-containing protein 3), an inflammasome-forming pattern recognition receptor. In conclusion, cerebral pericytes and endothelial cells mutually activate each other in inflammation.

## 1. Introduction

An increasing body of evidence suggests that vascular cells, namely cerebral endothelial cells (CECs) and pericytes, participate in and modulate inflammatory reactions of the central nervous system (CNS). Endothelial activation and dysfunction are key elements of neuroinflammation in several cerebral pathologies, including neurodegeneration, infection, stroke, and also aging [1]. Moreover, inflammatory responses of the neurovascular unit (NVU) can be among the first alterations in several cerebral pathologies. Vascular dysfunction may precede cognitive decline and neurodegenerative changes in Alzheimer’s disease (AD), whereas cerebral amyloid angiopathy is one of the hallmarks of the disease [2]. Increased permeability of the blood–brain barrier (BBB), enhanced expression of endothelial adhesion molecules, and consequent diapedesis of different leukocyte subsets complete the picture in both AD [3] and other neuroinflammatory conditions such as stroke [4] or infections [5].

Several molecular mechanisms are involved in neurovascular inflammatory reactions. CECs are targets of proinflammatory cytokines [6] leading to disruptive or nondisruptive BBB changes [7].

Disruptive changes comprise degradation of the glycocalyx, endothelial apoptosis, and, most importantly, impairment of the tight junctions (TJs) [8]. TJs are critical elements of the defense lines of the BBB [9]. Transmembrane proteins of the TJs (occludin, claudin-5, and others) linked to plaque proteins (e.g., zonula occludens/ZO-1 and -2) seal the paracellular cleft [10]. High claudin-5 content of brain endothelial TJs determines a high degree of impermeability to ions, which results in a transendothelial electrical resistance (TEER) on the two sides of the endothelial layer [11]. Disruption of TJs leads to TEER decrease, which can be monitored in vitro.

Among nondisruptive changes induced by inflammatory mediators, increased expression of cell adhesion molecules and release of cytokines are the most important. CECs are able to secrete interleukin/IL-1α, IL-6, IL-10, TNF-α and granulocyte–macrophage colony-stimulating factor (GM-CSF), partly in a polarized manner: secreting IL-6 and IL-10 (but not GM-CSF and TNF-α) preferentially into the apical direction, but responding more robustly to lipopolysaccharide (LPS) challenge coming from the basolateral side [12]. Beside CECs, brain pericytes can also upregulate cell adhesion molecules and secrete different cytokines and chemokines (IL-6, IL-8, CCL2, CXCL1, CXCL2, CXCL3, and macrophage inflammatory protein/MIP-1α) under inflammatory conditions [13,14] and contribute to the maintenance of the brain–immune interface [15].

In addition, both CECs and pericytes express pattern recognition receptors (PRRs), including Toll-like and NOD-like receptors (TLRs and NLRs), and they are able to activate inflammasomes [16,17,18]. Inflammasomes are multiprotein intracellular complexes composed of NLRs, adaptor proteins, and caspases, which, upon detection of microbial- or host damage-induced molecular patterns in the extracellular space, induce the activating cleavage of highly potent proinflammatory cytokines such as IL-1β and IL-18 [19]. Importantly, inflammasome formation needs separate signals for priming (i.e., upregulation of the expression of inflammasome components) and activation (i.e., assembly of the components to activate effector caspases). This latter step requires the ASC adaptor protein (apoptosis-associated speck-like protein containing a CARD/caspase activating and recruitment domain), which, upon NLR-induced polymerization, provides platforms for the activation of caspase-1. During this process, ASC assembles into a speck, which—beside secretion of active IL-1β—is considered a readout for inflammasome activation [20]. In addition to canonical inflammasomes, noncanonical inflammasomes have also been described; their activation depends on LPS reaching the cytoplasm of the cells [21]. Here, the effectors are caspase-4/5 in humans and caspase-11 in mice, which induce pyroptosis (an inflammatory form of programmed cell death), as well as IL-1β and IL-18 release through secondary activation of the NLRP3 inflammasome [22].

We have previously shown that CECs can activate inflammasomes through the canonical pathway [17], whereas the canonical inflammasome activation pathway was found to be inactive in brain pericytes. However, pericytes can respond to bacterial infection with noncanonical inflammasome activation [18].

Several questions remain unanswered. First, whether CECs are able to respond to bacteria with inflammasome activation and whether the noncanonical inflammasome pathway can be activated in these cells has not yet been investigated. Moreover, we wanted to characterize inflammasome activation in response to apical/luminal versus basolateral/abluminal stimuli. Separated by TJs, the apical/luminal membrane faces the blood, whereas the basolateral/abluminal side corresponds to the brain side. It is therefore important to understand whether CECs sense inflammatory signals coming from both the blood and the brain.

In addition, we addressed the question whether CECs and pericytes communicate with each other to propagate inflammasome-dependent signals. Notably, cerebral pericytes and endothelial cells have unique secretome profiles in response to IL-1β, a cytokine secreted principally after inflammasome activation [23]. Here, we aimed to understand whether activation of inflammasomes in CECs or pericytes activates the other cell type to propagate the inflammatory signal from endothelial cells to pericytes (i.e., from the blood to the brain) and from pericytes to the endothelium (i.e., from the CNS toward the periphery).

## 2. Results

### 2.1. Characterization of Inflammasome Activation in CECs

**Figure 1:**
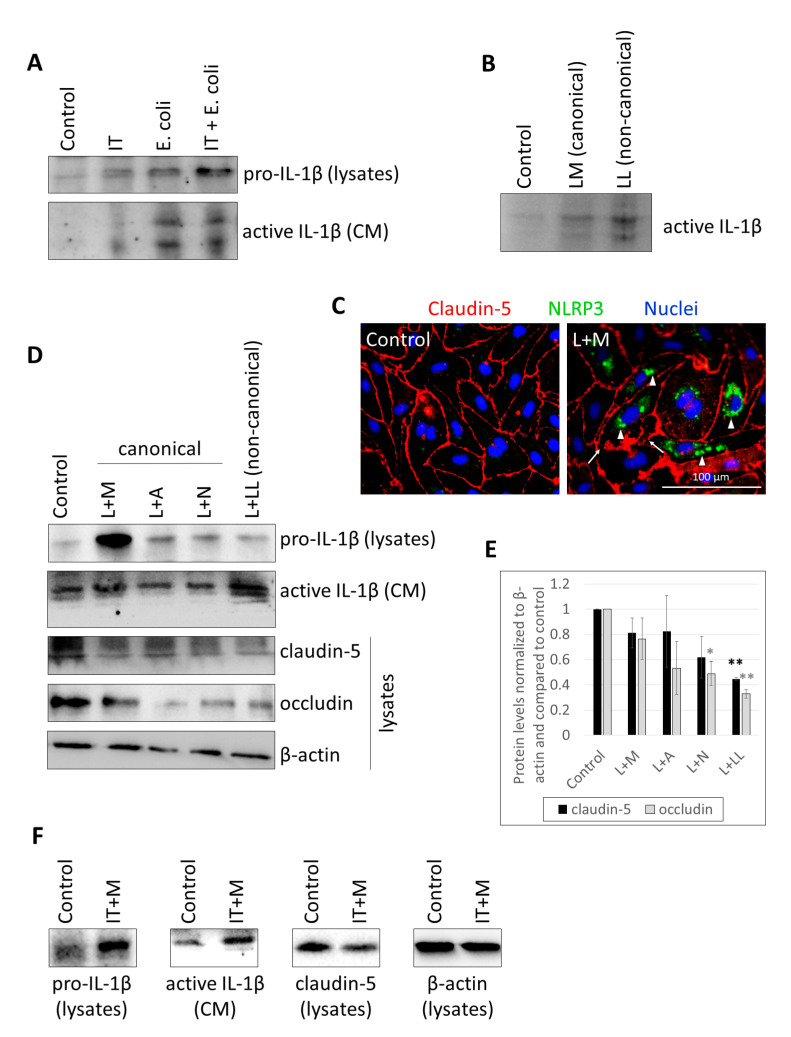
CECs secrete active IL-1β in response to *E. coli* infection, canonical and noncanonical inflammasome activation. (**A**) D3 cells were treated with 1000 U/mL IFN-γ and 10 ng/mL TNF-α (IT), 10^7^ CFU/mL *E. coli* or IFN-γ, TNF-α and *E. coli* (IT + *E. coli*) for 24 h. Cell lysates and culture media (CM) were collected for IL-1β Western blot. (**B**) D3 cells were treated with 1 µg/mL LPS and 100 µg/mL MDP (LM) or 2 µg/mL LPS and Lipofectamine (LL) for 24 h. Culture media were collected for IL-1β Western blot. (**C**) PBECs were primed with 1 µg/mL LPS and activated with 100 µg/mL MDP (L + M). Representative immunofluorescence images of claudin-5 and NLRP3 are shown. Nuclei are stained with Hoechst 33342. Arrows show TJ disruption. Arrowheads indicate perinuclear accumulation of NLRP3. (**D**) Representative IL-1β and TJ protein Western blots from D3 cells treated with 1 µg/mL LPS priming and activation with 100 µg/mL MDP (L + M), 5 mM ATP (L + A), 10 µM nigericin (L + N), or 2 µg/mL LPS and Lipofectamine (L + LL). (**E**) Quantification of TJ protein expression (average +/- SEM, *N* = 3, * *p* < 0.05, ** *p* < 0.01, as assessed by ANOVA and Bonferroni’s post hoc test). (**F**) Representative IL-1β and claudin-5 Western blots from D3 cells primed with 1000 U/mL IFN-γ + 10 ng/mL TNF-α and activated with 100 µg/mL MDP (IT + M).

Previously, we have described NLR expression in CECs and demonstrated canonical inflammasome activation in these cells [17]. Here, we aimed to further characterize inflammasome activation in CECs; therefore, we treated brain endothelial cultures with different agents, which induce inflammasome activation. We observed that beside LPS + MDP (muramyl dipeptide), other priming and activation stimuli also lead to active IL-1β release. Using IFN-γ + TNF-α and/or *E. coli*, we detected both the priming effect (i.e., upregulation of pro-IL-1β expression in the cells) and the result of inflammasome activation (i.e., secretion of cleaved IL-1β into the culture medium) (Figure 1A, Supplementary Figure S1).

In addition, we aimed to understand whether the noncanonical pathway could also be induced in CECs beside canonical inflammasome activation that we reported previously. Therefore, LPS was transferred to the cytosol of CECs with Lipofectamine, resulting in an even more robust active IL-1β secretion than that observed in response to the classical canonical activator LPS and MDP combination (Figure 1B, Supplementary Figure S2A). Lipofectamine alone did not induce IL-1β upregulation (Supplementary Figure S2B). If priming and activating stimuli were separated in time (LPS priming was applied for 24 h, while the activator MDP was given in the last 6 h), NLRP3 upregulation and assembly was observed together with disruption of the TJs (Figure 1C). Inflammasome activation was further observed with LPS priming followed by administration of a diverse set of canonical activation stimulators and also with LPS priming followed by cytosolic LPS-induced activation of the noncanonical pathway (Figure 1D, Supplementary Figure S3). The strongest priming effect was seen with the LPS + MDP combination, while the highest level of active IL-1β was detected in the culture media of noncanonically activated cells. In parallel, in response to the barrier disruptive effect of the proinflammatory milieu, the amount of TJ proteins decreased (Figure 1D,E). Similarly, if IFN-γ + TNF-α priming was used together with MDP-induced inflammasome activation, upregulation of IL-1β expression, and secretion of active IL-1β were accompanied by decrease in claudin-5 levels (Figure 1F, Supplementary Figure S4).

**Figure 2:**
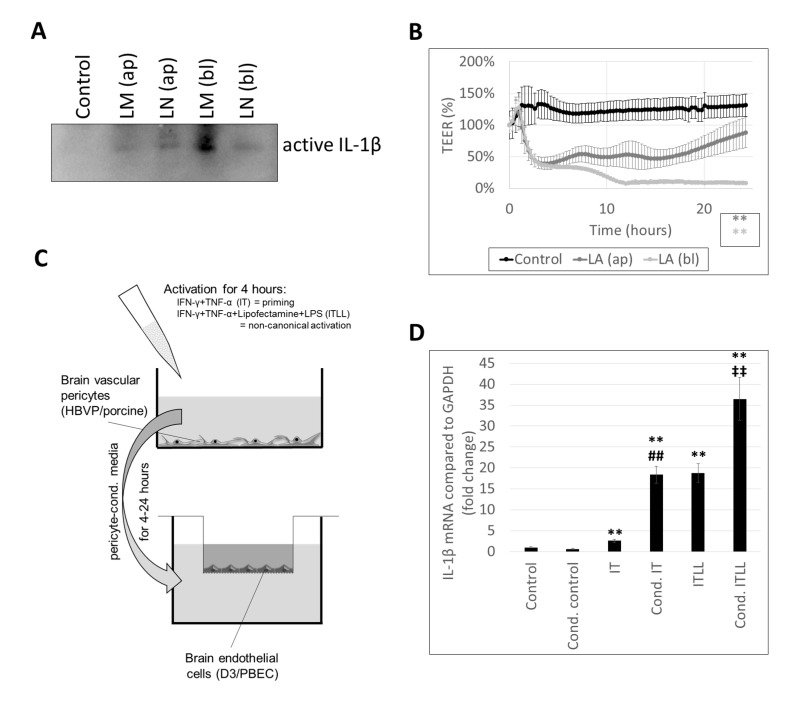
CECs respond to both apical and basolateral inflammatory stimuli. (**A**) PBECs were treated with 1 µg/mL LPS and 100 µg/mL MDP (LM) or 1 µg/mL LPS and 10 µM nigericin (LN) for 24 h from the apical (ap) or the basolateral (bl) compartment. Media were collected from the basolateral compartment for IL-1β Western blot. (**B**) PBECs were treated with 1 µg/mL LPS and 5 mM ATP (LA) for 24 h from the apical (ap) or the basolateral (bl) compartment. TEER was followed with the CellZscope instrument for 24 h (average +/- SD, *N* = 3, ** *p* < 0.01 compared to control, as assessed by comparing areas under curve with ANOVA and Bonferroni’s post hoc test). (**C**) Experimental setup for studying the effect of activated pericytes on brain endothelial cells. (**D**) D3 cells were treated from the basolateral side for 4 h with conditioned media collected from HBVPs exposed to inflammatory stimuli. Graph shows IL-1β mRNA levels (average +/- SD), *N* = 3, ** *p* < 0.01 compared to control, **^##^** *p* < 0.01 compared to IT, **^‡‡^** *p* < 0.01 compared to ITLL, as assessed by ANOVA and Bonferroni’s post hoc test.

As a result of the fence function of TJs, CECs are highly polarized cells, having an apical and a basolateral membrane with distinct lipid and protein composition. Therefore, we tested whether inflammasome activation can be induced by stimuli coming from both the apical and the basolateral directions. We observed active IL-1β secretion not only in response to apical activation, but also when LPS and inflammasome activators were administered to the basolateral chamber of the cells cultured in filter inserts (Figure 2A, Supplementary Figure S5). Basolateral stimulation resulted in similar or even higher amounts of secreted IL-1β (Figure 2A, Supplementary Figure S5) and stronger impairment of the TJs, as indicated by the sharper and more prolonged decrease in TEER in response to basolaterally added LPS + ATP in comparison to the apical administration of the same combination (Figure 2B).

### 2.2. Activation of CECs by Pericytes Exposed to Inflammasome Activator Stimuli

Since there was an explicit response from CECs to basolateral inflammatory stimuli, we next aimed to understand whether CECs react in a similar manner to inflammatory stimulation coming from cells located close to their basolateral surface. Pericytes are perivascular cells, sharing the basement membrane with CECs, coming in direct contact with their basolateral membrane. To test whether pericytes exposed to inflammatory stimuli may activate inflammatory pathways in CECs, we designed an in vitro setup (Figure 2C). We first induced noncanonical inflammasome activation in pericytes, collected their culture media containing released inflammatory factors, and added those to the basolateral chamber of CECs cultured on filter inserts. As controls, we applied control pericyte-conditioned media (i.e., media collected from nonstimulated pericytes) and nonconditioned media. Altogether, six conditions were tested. Control endothelial cells received serum-free medium (not conditioned on pericytes and containing no inflammatory mediators). Cond. control cells were given pericyte-conditioned control medium. IT-treated CECs received serum-free medium containing 1000 U/mL IFN-γ and 10 ng/mL TNF-α; for ITLL-treated cells, the medium also contained Lipofectamine-bound LPS. Cond. IT and cond. ITLL conditions mean that endothelial cells received pericyte-conditioned media collected from pericytes treated with IT or ITLL, respectively. All nonconditioned media given to CECs were kept in the same incubator as the pericytes for the time until pericytes were stimulated with IT and ITLL. Using this complex setup, we could clearly differentiate between the effect of inflammatory factors used to stimulate pericytes and those released by these cells upon CECs.

Using this setup, we first demonstrated that cond. IT media induce a significantly higher upregulation of IL-1β mRNA expression in CECs than IT stimulation. This is probably not the consequence of IL-1β secreted by pericytes, but of other inflammatory mediators, since pericytes are only able to activate the noncanonical inflammasome pathway. ITLL treatment (i.e., noncanonical inflammasome activation) resulted in even higher IL-1β levels than IT; the highest IL-1β expression was seen in CECs exposed to pericytes in which the noncanonical inflammasome pathway was induced (cond. ITLL) (Figure 2D). These results show that inflammasome activation in pericytes transmits signals to CECs to induce the inflammasome pathway.

**Figure 3:**
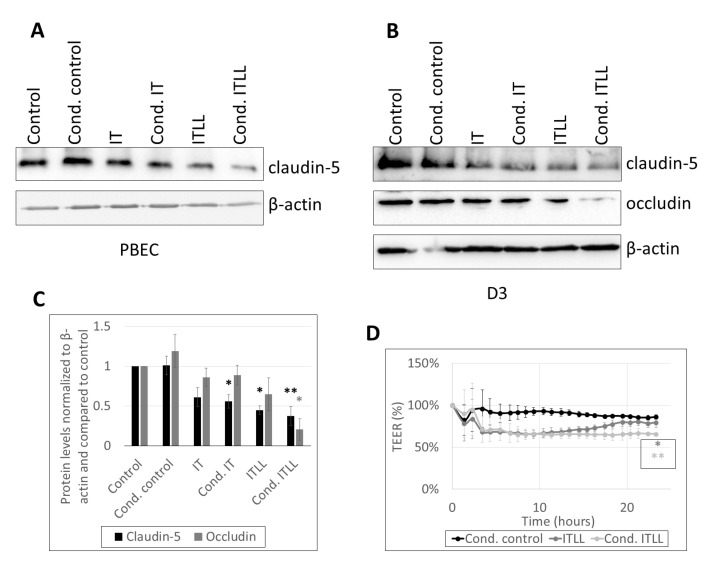
Inflammatory stimuli released by pericytes alter barrier properties of CECs. (**A**) Western blot analysis of PBECs treated from the basolateral side for 24 h with conditioned media collected from porcine brain pericytes exposed to inflammatory stimuli. (**B**) Western blot analysis of D3 cells treated from the basolateral side for 24 h with conditioned media collected from HBVPs exposed to inflammatory stimuli. (**C**) Quantitative analysis of data shown in **B** (average +/- SD, *N* = 3, * *p* < 0.05, ** *p* < 0.01, as assessed by ANOVA and Bonferroni’s post hoc test). (**D**) PBECs were treated from the basolateral side with ITLL or conditioned media of porcine brain pericytes exposed to ITLL (Cond. ITLL). TEER was followed with the CellZscope instrument for 24 h (average +/- SD, *N* = 2, * *p* < 0.05, ** *p* < 0.01 compared to control, as assessed by comparing areas under curve with ANOVA and Bonferroni’s post hoc test).

These pericyte-transmitted signals caused the impairment of TJs of CECs both in the porcine and in the human model. A stronger decrease in the amount of claudin-5 was detected in response to ITLL (noncanonical inflammasome activation) than in response to IT; however, the largest reduction was in cond. ITLL-treated cells (Figure 3A–C, Supplementary Figures S6 and S7), underlining again the role of factors secreted by pericytes in response to the noncanonical inflammasome activation. Similarly, loss of occludin was the highest in cond. ITLL-treated cells (Figure 3B,C). In parallel, drop in the TEER lasted longer in cells exposed to cond. ITLL media than ITLL only (Figure 3D).

**Figure 4:**
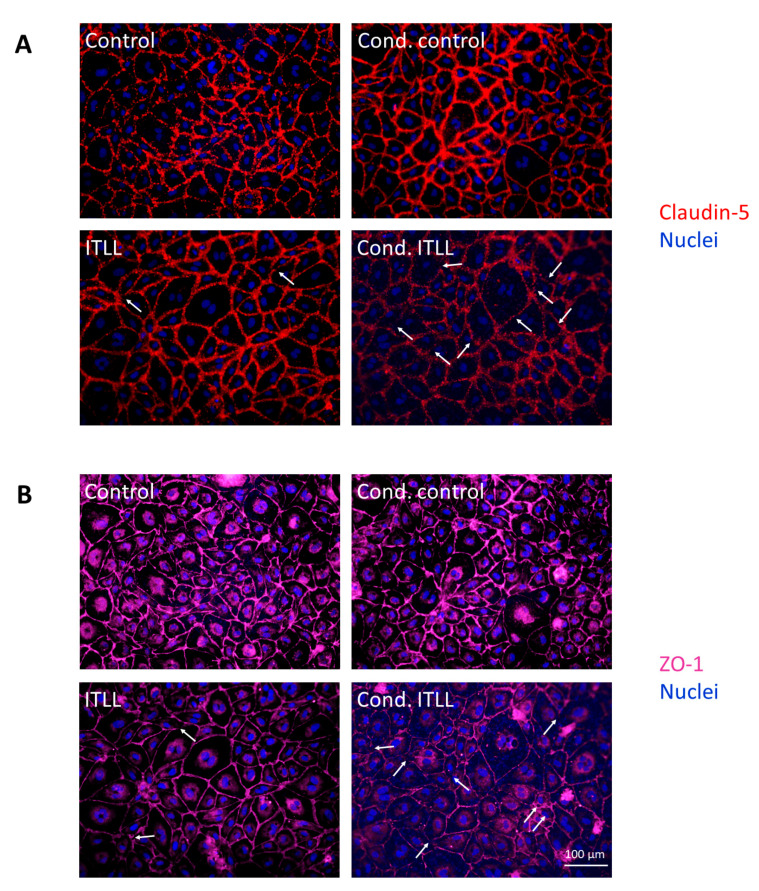
Inflammatory stimuli released by pericytes disrupt TJs of CECs. PBECs were treated from the basolateral side for 24 h with conditioned media collected from porcine brain pericytes exposed to noncanonical inflammasome activation. Representative immunofluorescence images of claudin-5 (**A**) and ZO-1 (**B**) TJ proteins are shown. Nuclei are stained with Hoechst 33342. Arrows show TJ disruption.

We next examined TJs using fluorescence microscopy and observed decreased signal intensity and discontinuities in both occludin and ZO-1 in cells exposed to cond. ITLL media (Figure 4). All of these results show that CECs detect factors secreted by pericytes in which the noncanonical inflammasome pathway is activated and respond with inflammatory activation and impairment of the barrier properties.

### 2.3. Activation of Brain Pericytes by CECs Exposed to Inflammasome Activator Stimuli

**Figure 5:**
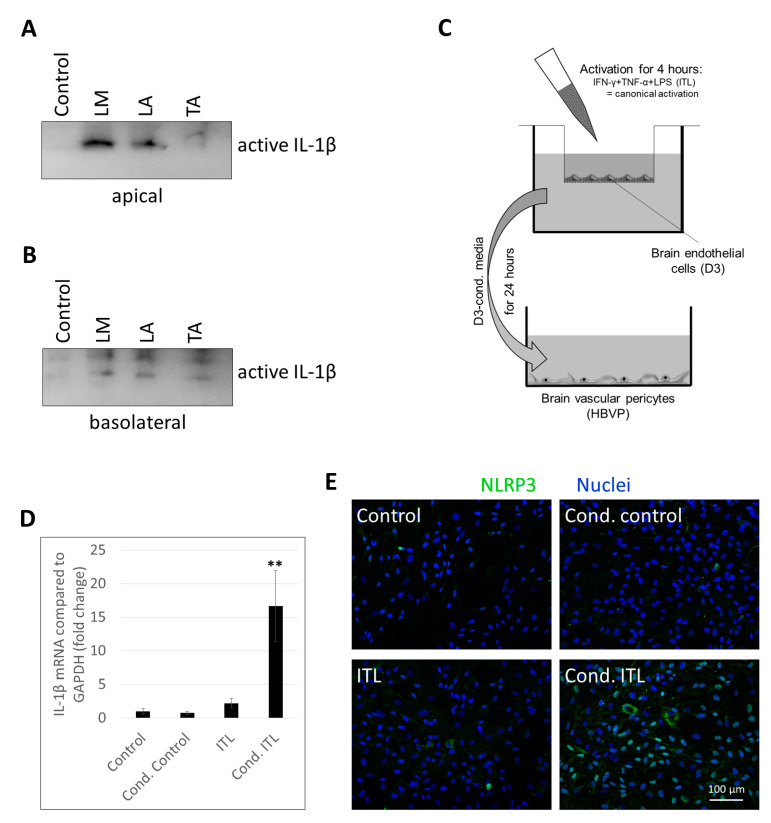
Activated CECs secrete inflammatory mediators into both apical and basolateral directions. (**A,B**) PBECs were treated with 1 µg/mL LPS and 100 µg/mL MDP (LM), 1 µg/mL LPS and 5 mM ATP (LA), or 10 ng/mL TNF-α and 5 mM ATP (TA) for 24 h from the apical compartment. Media were collected from both the apical (**A**) and basolateral (**B**) compartments for IL-1β Western blot. (**C**) Experimental setup for studying the effect of activated brain endothelial cells on pericytes. (**D**) HBVP cells were treated with conditioned media collected from D3 cells exposed to inflammatory stimuli. Graph shows IL-1β mRNA levels (average +/- SD), *N* = 3, ** *p* < 0.01 compared to control, as assessed by ANOVA and Bonferroni’s post hoc test. (**E**) HBVP cells were treated for 24 h with conditioned media collected from the basolateral side of D3 cells exposed to inflammatory stimuli from the apical side. Representative NLRP3 immunofluorescence images are shown. Nuclei are stained with Hoechst 33342.

Our next question was whether transmission of inflammatory signals from one cell to the other is mutual in cerebral pericytes and endothelial cells. Therefore, we first stimulated CECs from the apical direction to test whether cytokine secretion occurs only in the direction from where the activation comes from (i.e., the apical compartment) or into both the apical and basolateral sides. We observed that several different inflammasome activator stimuli applied in the apical chamber induced secretion of active IL-1β into both directions (Figure 5A,B, Supplementary Figures S8 and S9). This indicates that by sensing inflammatory signals from the apical/blood side, CECs may release cytokines toward the basolateral/brain side as well to activate other cells of the NVU, particularly pericytes.

In order to test this hypothesis, we constructed an in vitro model (Figure 5C). CECs cultured on filter inserts received ITL treatment from the apical compartment. After 4 h, conditioned media were collected from the basolateral chamber and added to brain pericytes. Cond. control cells received media from the basolateral compartment of nontreated CECs. In addition, nonconditioned media were also used containing or not containing ITL (ITL and control, respectively), which were collected from the basolateral side of cell-free culture inserts kept in the same conditions as CECs during the 4 h of stimulation.

As previously shown [18], the canonical inflammasome pathway is not active in brain pericytes; therefore, there was no significant upregulation of IL-1β mRNA in response to ITL treatment. In contrast, when pericytes were challenged with conditioned media of CECs exposed to ITL, a robust induction of IL-1β transcription was detected (Figure 5D). Similarly, in response to ITL, NLRP3 expression was induced only in a few cells. On the other hand, cond. ITL-challenged cells unequivocally upregulated NLRP3, and the protein appeared both in the nuclei of the cells and in the cytoplasm (Figure 5E). This suggests that upon sensing of inflammatory signals, CECs release inflammatory factors both toward the blood and the brain sides and activate pericytes among other cells.

## 3. Discussion

Neuroinflammation is a common characteristic of the majority of neurological disorders, but also of aging and of some systemic diseases [24,25,26]. Besides infiltrating cells of the innate and adaptive immune systems, resident cells of the brain also participate in the initiation and propagation of inflammatory reactions. Under inflammatory conditions, all cells of the NVU may become activated, including CECs, pericytes, glial cells (microglia and astrocytes) [25], and even neurons [27]. Notably, implication of cerebral vessels has two main aspects: BBB opening and cytokine secretion.

Among proinflammatory cytokines, IL-1β is one of the most potent, being released primarily in an inflammasome-dependent manner [28]. We have previously shown that both CECs and brain pericytes are able to activate inflammasomes, the latter only through the noncanonical pathway upon incorporation of LPS or bacteria [18]. Our present results show that several different priming and activator stimuli, including bacteria, can induce inflammasomes in CECs and that both the canonical and the noncanonical pathways are functional in these cells. Importantly, all types of proinflammatory activators, including cytokines and pathogen-associated molecular patterns (PAMPs), such as LPS, MDP, or nigericin, reduced TJ protein levels and compromised barrier properties of CECs. This is in line with previous observations showing co-occurrence of disruptive and nondisruptive BBB changes in inflammatory conditions [7]. Notably, the most severe junctional disruption was observed upon noncanonical inflammasome activation.

An important and largely unexplored aspect of neuroinflammation is information exchange and mutual activation in different cell types of the brain. Crosstalk among cells of the NVU is critical for the maintenance of a proper BBB that protects the brain from potentially harmful substances but also provides it with the necessary nutrients. However, endothelial–pericyte communication needs special attention because endothelial cells are forming the interface between the CNS and the periphery [29]. CECs may receive information from pericytes to alert other brain cells and cells of the immune system about inflammatory conditions in the CNS. To test this hypothesis, we first demonstrated that CECs detect inflammasome activators not only from the apical/blood/luminal side but also from the basolateral/brain/abluminal side and respond with active IL-1β secretion and junctional disruption. Moreover, inflammasome-activating agents could result in higher IL-1β secretion (as shown for LPS + MDP) and also a higher drop in the TEER (as shown for LPS + ATP) when administered basolaterally. Intense response of CECs to abluminally administered LPS has already been demonstrated for non-inflammasome-dependent cytokines in an in vitro mouse model [12]. Robust basolateral cytokine secretion in response to abluminal, but not to luminal LPS was observed in a triple coculture model of the BBB cells as well [30]. Altogether, present and previous data show that abluminal LPS is generally more robust in inducing cytokine secretion than luminal stimulation for a diverse range of cytokines (IL-1β, IL-6, IL-10, IL-12, MIP-1α and MIP-1β).

As a next step, we showed that CECs are also able to detect basolateral inflammatory signals secreted by pericytes in response to inflammasome activation. CECs responded with disruptive and nondisruptive changes to pericyte-derived inflammatory mediators as well as to bacterial signals. Indeed, intercellular crosstalk among cells of the NVU has been shown to determine a unique pattern of cytokine secretion dependent on both external activation and stimuli coming from surrounding cells [30]. Moreover, pericytes were also shown to communicate with microglia and induce marked IL-1β mRNA expression in these cells in response to TNF-α stimulation [14]. These data indicate that an inflammatory reaction in the brain readily alerts all cells of the NVU, which activate each other. Transmission of inflammatory signals to CECs may eventually be translated to the periphery as well to possibly attract immune cells.

On the other hand, peripheral signals might also be translated to the brain. Being the first sensors of blood-borne molecules and cells, CECs may transmit systemic inflammatory signals to pericytes, with whom they form direct contacts [31]. Importantly, activation of cerebral endothelial IL-1 receptor 1 was shown to mediate sickness behavior, neutrophil infiltration, microglia activation, and neurogenesis in mice [32].

We observed that inflammasome activation induced by apical stimulation of CECs resulted in secretion of active IL-1β into both the blood and the brain sides. Moreover, through this setup, NLRP3 upregulation was observed in pericytes, showing propagation of the inflammatory signal from the periphery to the brain. Nevertheless, it has been previously suggested that interactions between peripheral and central inflammasome-mediated pathways may contribute to neuroinflammatory conditions [25]. In this respect, association of type 2 diabetes and AD [33], linkage between intestinal dysfunction and neurodegeneration through the gut–brain axis [34], and inflammaging [35] are a few examples to be considered. In addition, CECs activated with luminal LPS were shown to release cytokines that stimulated pericytes, eventually leading to enhanced HIV (human immunodeficiency virus) transport [36], further underlining the importance of inflammatory signal propagation from the periphery to the brain.

In conclusion, we provide direct evidence of transmission of inflammatory and especially inflammasome-dependent signals by endothelial cells of the BBB from the brain to the periphery and vice versa. Mutual activation of CECs and brain pericytes may result in cytokine secretion and BBB disruption to activate surrounding cells, to inform the immune system about neuroinflammation, and to propagate systemic inflammation to the CNS.

## 4. Materials and Methods

### 4.1. Isolation and Culture of Brain Endothelial Cells and Pericytes

Porcine brain endothelial cells (PBECs) were isolated from the grey matter of young female Vietnamese pot-bellied pigs, as described previously [37], selected with 4 µg/mL puromycin (Merck-Sigma, Darmstadt, Germany) for 4 days and cultured in Dulbecco’s modified Eagle’s medium and Ham’s F-12 (DMEM/F12; Gibco, Thermo Fisher Scientific, Waltham, MA, USA) supplemented with 10% plasma-derived serum (PDS; First Link, Birmingham, UK) and growth factors. Porcine brain pericytes were obtained from the same fraction by seeding the microvessels onto noncoated surfaces in DMEM (Gibco, Thermo Fisher Scientific) and 10% fetal bovine serum (FBS; Gibco, Thermo Fisher Scientific). D3 cells (hCMEC/D3; obtained from Prof. P.-O. Couraud, INSERM, France) were maintained in rat-tail collagen coated dishes in EBM-2 medium supplemented with EGM-2MV bullet kit (Lonza, Bazel, Switzerland). Human brain vascular pericytes (HBVPs; ScienCell, Carlsbad, CA, USA) were cultured in PM medium (ScienCell) and 5% FBS.

### 4.2. Inflammatory Activation of Brain Endothelial Cells and Pericytes

In order to understand whether bacterial stimuli induce inflammasome activation in CECs, D3 cells were treated with 10^7^ CFU/mL *E. coli* or 1000 U/mL interferon/IFN-γ + 10 ng/mL tumor necrosis factor/TNF-α (IT) + *E. coli* (IT + *E. coli*) for 24 h. Canonical inflammasome priming and activation were induced with the following treatments: 1000 U/mL IFN-γ + 10 ng/mL TNF-α + 1 µg/mL LPS (ITL), 1 µg/mL LPS + 100 µg/mL MDP (LM), 1 µg/mL LPS + 5 mM adenosine triphosphate/ATP (LA), 1 µg/mL LPS + 10 µM nigericin (LN) or 10 ng/mL TNF-α + 5 mM ATP (TA) for 4 or 24 h. In certain experiments, LPS or IFN-γ + TNF-α priming was applied for 24 h, while activator stimuli were given in the last 6 h (L+M, L+A, L+N, and IT+M, respectively). Noncanonical inflammasome activation was performed using 2 µg/mL LPS + Lipofectamine 2000^®^ (LL) or 1 µg/mL LPS priming for 24 h and 2 µg/mL LPS + Lipofectamine 2000^®^ applied in the last 6 h (L+LL). Lipofectamine 2000^®^ was used in a final concentration of 5 μL/well (in a 6-well plate) and was preincubated with LPS for 15 min. Canonical and noncanonical inflammasome activation of PBECs and D3 cells was performed in serum-free DMEM. Serum-free PM medium was used for the inflammatory activation of HBVP cells. IFN-γ, TNF-α, LPS (from *E. coli*, strain 0111:B4), and ATP were purchased from Merck-Sigma. MDP was obtained from Bachem, Bubendorf, Switzerland, Lipofectamine^®^2000 from Thermo Fisher Scientific. Nigericin was obtained from Tocris/Bio-Techne, Minneapolis, MN, USA.

### 4.3. Coculture Systems for the Study of Endothelial–Pericyte Communication

For studying pericyte-to-endothelial communication, brain pericytes were cultured in 6 cm diameter cell culture dishes until they reached almost complete confluency. Culture medium was replaced with IT- or ITLL-containing PM medium, while control cells received control medium. In parallel, two cell-free culture dishes were placed into the CO_2_ incubator for the same time, one containing IT/ITLL in PM medium, the other with control medium. After 4 h, media were collected and kept at 4 °C for up to 24 h. Brain endothelial cells were cultured in filter inserts of 24 mm diameter (Transwell Clear, pore size: 0.4 µm; Costar-Corning, Corning, NY, USA). Collected media were added to the abluminal compartment of confluent brain endothelial cells in a ratio of 1:1 and left for 4–24 h (Figure 2C).

For studying endothelial-to-pericyte communication, D3 cells were cultured in filter inserts and exposed to ITL treatment from the luminal side using serum-free PM medium. After 24 h, media were collected from the abluminal compartment and added for 4 h to HBVPs in a ratio of 1:1 (Figure 5C).

### 4.4. Measurement of Transendothelial Electrical Resistance (TEER)

PBECs were plated into fibronectin/collagen-coated filter inserts of 12 mm diameter (Costar-Corning Transwell Clear, pore size: 0.4 µm). After reaching confluency, endothelial monolayers were supplied with 550 nM hydrocortisone, 250 μM CPT-cAMP and 17.5 μM RO-201724 and placed into the CellZscope instrument (nanoAnalytics, Münster, Germany). TEER was followed continuously. Treatments were applied after approx. 24 h, when TEER reached plateau. TEER was further monitored for 48 h.

### 4.5. Sample Preparation and Western Blot

Culture media were collected, treated with 1 mM EDTA, and centrifuged at 2500 × *g* at 4 °C for 1 min to settle cellular debris. Protein content of cell culture media samples was precipitated with the methanol–chloroform method, as described previously [18]. Cells were lysed in ice-cold RIPA buffer (20 mM Tris-HCl pH = 7.4, 150 mM NaCl, 1% v/v Triton X-100, 1% m/v Na-deoxycholate, 1 mM Na_3_VO_4_, 10 mM NaF, 0.1% m/V SDS, 1 mM EDTA and 1 mM Pefabloc), centrifuged at 9500 × *g* at 4 °C for 30 min. Protein concentration was determined with the bicinchoninic acid method (Santa Cruz Biotechnology, Dallas, TX, USA). Proteins were electrophoresed with standard denaturing SDS-PAGE procedures and blotted on polyvinylidene difluoride (Bio-Rad, Hercules, CA, USA) membranes. After blocking, the membranes were incubated with primary antibodies overnight, washed repeatedly, and incubated with the secondary antibodies. Antibodies used are shown in Table 1. The immunoreaction was visualized with the Bio-Rad Clarity Chemiluminescent Substrate in a ChemiDoc MP imaging system (Bio-Rad). Quantifications were performed using the ImageLab software version 5.2 (Bio-Rad).

### 4.6. Immunofluorescence and Fluorescence Microscopy

PBECs were cultured on filter inserts, whereas HBVPs were on glass coverslips. PBECs were fixed with 4% PFA for 10 min, permeabilized with 0.2% Triton X-100 for 20 min at room temperature, and blocked with 3% BSA for 60 min, all at room temperature. HBVPs were fixed with 100% methanol for 20 min at −20 °C, permeabilized with 0.2% Triton X-100 for 20 min, and blocked with 0.1% BSA in 0.2% Triton X-100-containing PBS for 15 min at room temperature. Primary antibodies (Table 1) were applied overnight at 4 °C. After thorough washing with PBS, secondary antibodies (Table 1) were added to the samples for 1 h at room temperature. Nuclei were counterstained with Hoechst 33,342 (Merck-Sigma). Cells were washed, rinsed, and then mounted in Fluoromount G (Thermo Fisher Scientific, Waltham, MA, USA). Distribution of the signal was studied with an epifluorescence microscope (Eclipse TE2000U; Nikon, Tokyo, Japan) connected to a digital camera (Spot RT KE; Diagnostic Instruments, Sterling Heights, MI, USA).

### 4.7. Real-Time PCR

Total RNA was isolated with the RNAqueous^®^-Micro Kit (Ambion, Thermo Fisher Scientific) according to the manufacturer’s instructions. Reverse transcription was done with the Maxima First Strand cDNA Synthesis Kit (Thermo Fisher Scientific). The qPCR reaction was performed using iTaq Universal SYBR Green Supermix (Bio-Rad) on an iQ5 thermocycler (Bio-Rad). The protocol consisted of 40 cycles (95 °C for 15 s, 61 °C for 30 s and 72 °C for 30 s). Primer sequences were described previously [17]. Quantification was performed with the ΔΔCt method using comparison to GAPDH levels.

## Figures and Tables

**Table 1:** Primary and secondary antibodies used.

Primary Antibody	Cat. No.	Application
anti-IL-1β g. polyclonal	AF-401-NA (R&D Systems/Bio-Techne)	WB: 1:500 in 1% BSA in TBS-T
anti-claudin-5 m. monoclonal	35-2500 (Thermo Fisher Scientific)	WB: 1:300 in TBS-T
		IF: 1:50 in 1% BSA in PBS
anti-occludin g. polyclonal	sc-8145 (Santa Cruz Biotechnology)	WB: 1:500 in TBS-T
anti-β-actin m. monoclonal	AC-15 (Merck-Sigma)	WB: 1:1000 in 1% BSA in TBS-T
anti-ZO-1 r. polyclonal	61-7300 (Thermo Fisher Scientific)	IF: 1:50 in 1% BSA in PBS
anti-NLRP3 g. polyclonal	GTX88190 (GeneTex, Hsinchu City, Taiwan)	IF: 1:200 in 1% BSA in PBS
**Secondary Antibody**	**Cat. No.**	**Application**
HRP-conjugated g. anti-mouse IgG (H + L)	610094 (BD Biosciences, San Jose, CA, USA)	WB: 1:3000 in TBS-T
HRP-conjugated g. anti-rabbit IgG (H + L)	7074 (Cell Signaling Technology)	WB: 1:4000 in TBS-T
Alexa Fluor^®^ 594 AffiniPure g. anti-mouse IgG (H + L)	115-585-003 (Jackson Immuno Research, Ely, UK)	IF: 1:600 in 1% BSA in PBS
Alexa Fluor^®^ 647 AffiniPure g. anti-rabbit IgG (H + L)	111-605-003 (Jackson Immuno Research)	IF: 1:600 in 1% BSA in PBS
Alexa Fluor^®^ 488 Cross-Adsorbed d. anti-goat IgG (H + L)	A-11055 (Thermo Fisher Scientific)	IF: 1:600 in 1% BSA in PBS

g. = goat, m. = mouse, r. = rabbit, d. = donkey, WB = Western blot, IF = immunofluorescence.

## Data Availability

The data presented in this study are available on request from the corresponding authors.

## Supplementary Materials

The following are available online at https://www.mdpi.com/article/10.3390/ijms22116122/s1, Figures S1–S9: uncropped Western blots.
