# Supplementary material for: Cerebral Pericytes and Endothelial Cells Communicate through Inflammasome-Dependent Signals
Source: Int J Mol Sci. 2021 Jun 6;22(11):6122. doi: 10.3390/ijms22116122 (PMC8201302; doi:10.3390/ijms22116122)
Supplement: Supplementary file 1 [file ijms-22-06122-s001.zip › ijms-1214006-supplementary.pdf]

# Supplementary figure S1

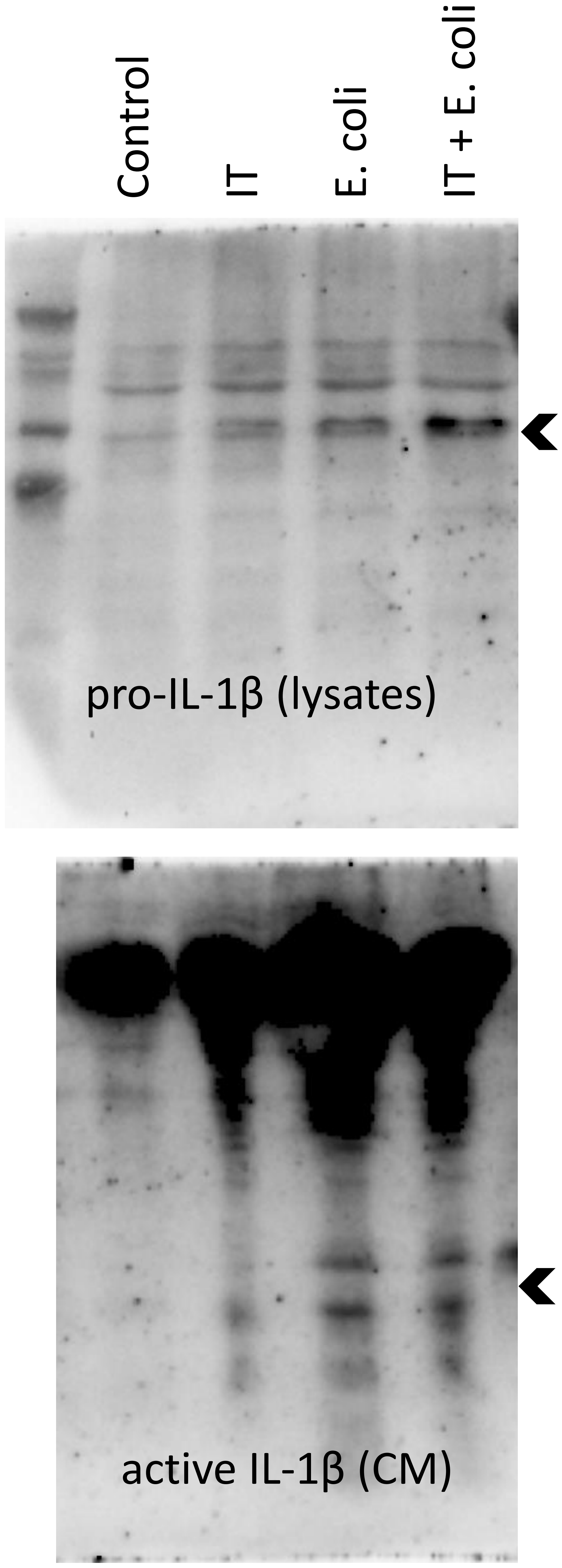


**Supplementary figure S1.** CECs secrete active IL-1β in response to *E. coli* infection. Uncropped blots of **Figure 1A**.

## Supplementary figure S2

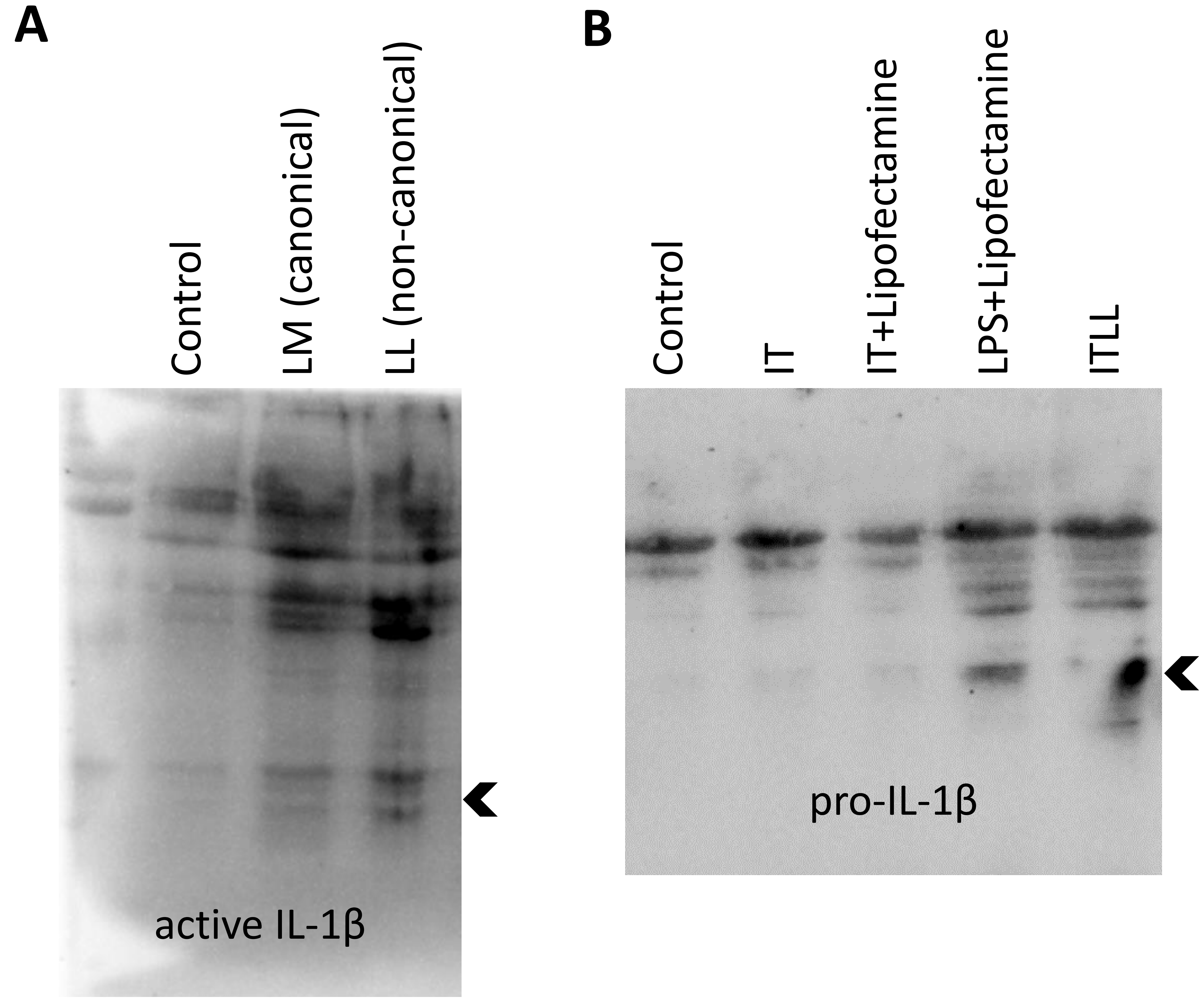


**Supplementary figure S2.** CECs secrete active IL-1β in response to canonical and noncanonical inflammasome activation.

(**A**) Uncropped blots of **Figure 1B**.

(**B**) Representative Western blot showing that Lipofectamine alone does not induce IL-1β upregulation. D3 cells were treated with:

- 1000 U/mL interferon/IFN-γ + 10 ng/mL tumour necrosis factor/TNF-α (IT)
- IT and Lipofectamine (5 µl/well in a 6-well plate)
- 2 µg/mL LPS and Lipofectamine
- IT + LPS + Lipofectamine (ITLL).

## Supplementary figure S3

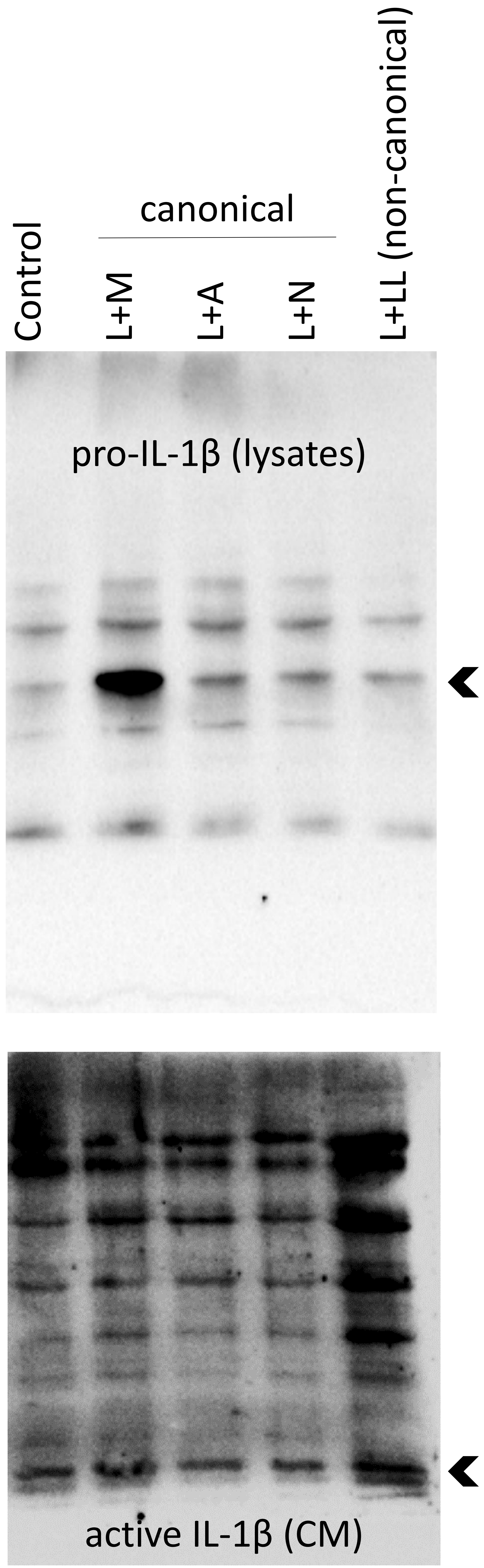


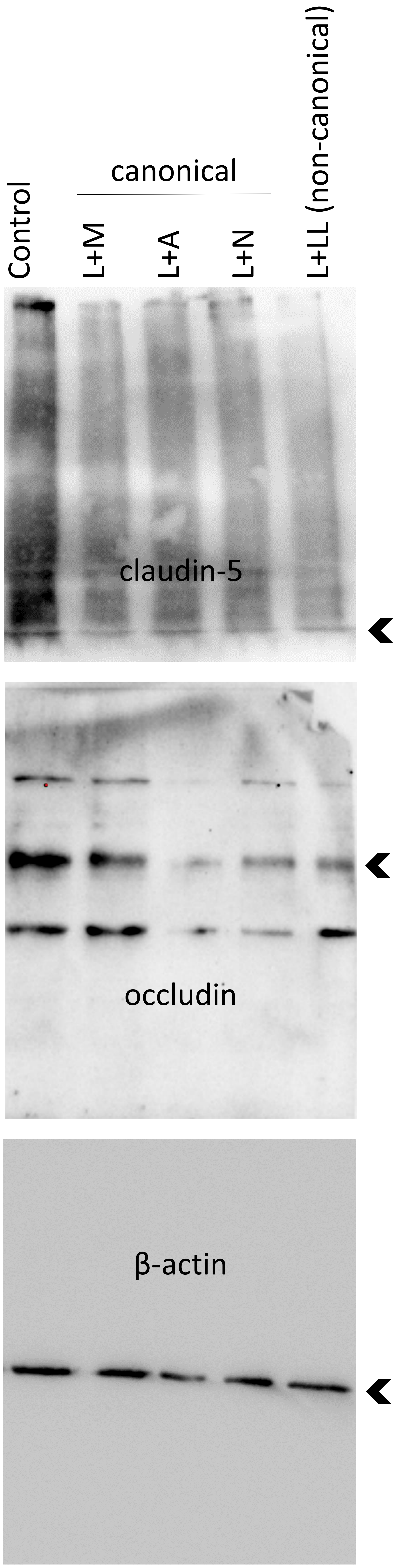


**Supplementary figure S3.** Canonical and noncanonical inflammasome activation induce active IL-1β secretion and TJ disruption in CECs.
Uncropped blots of **Figure 1D**.

## Supplementary figure S4

**A**

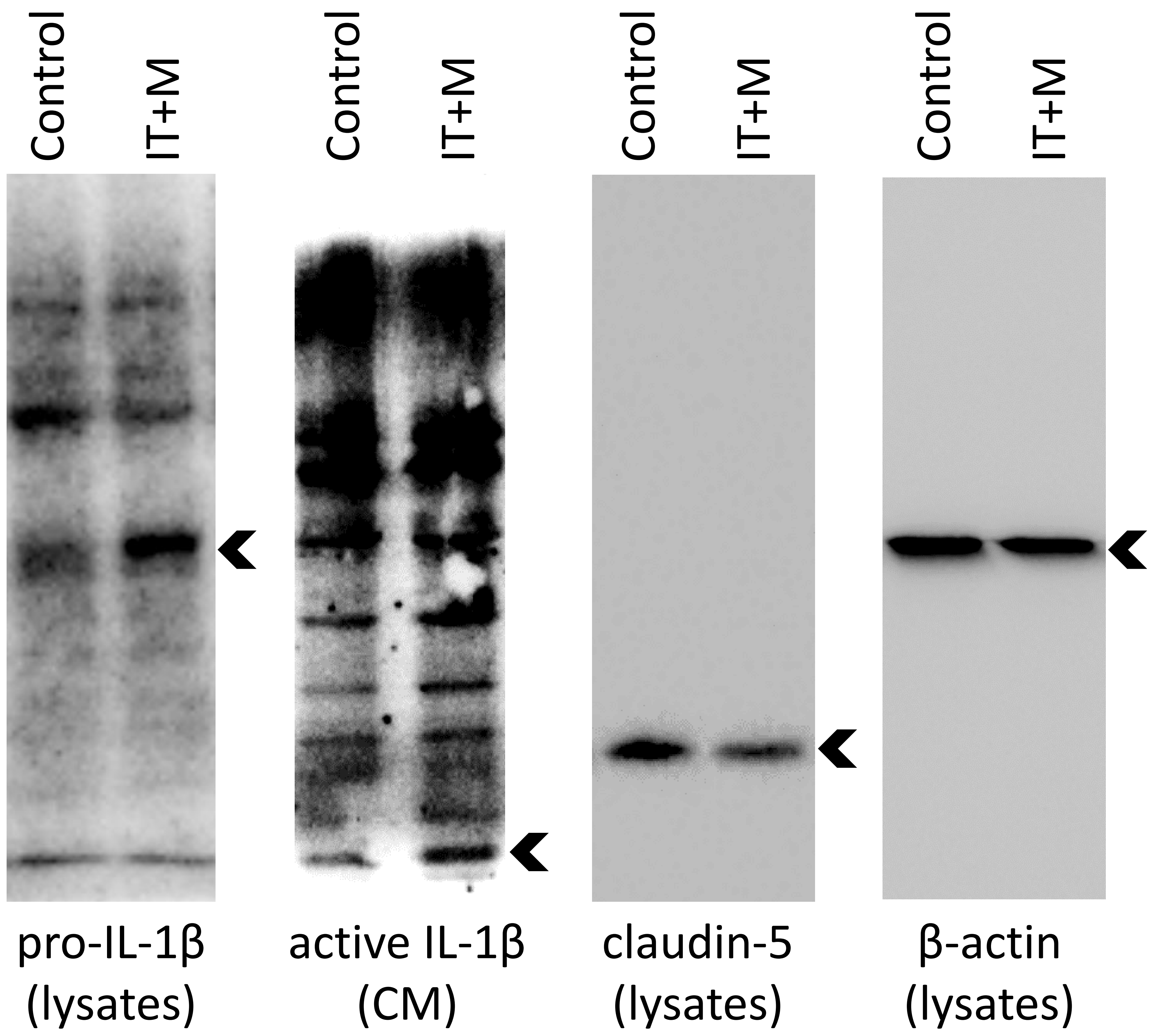


**B**

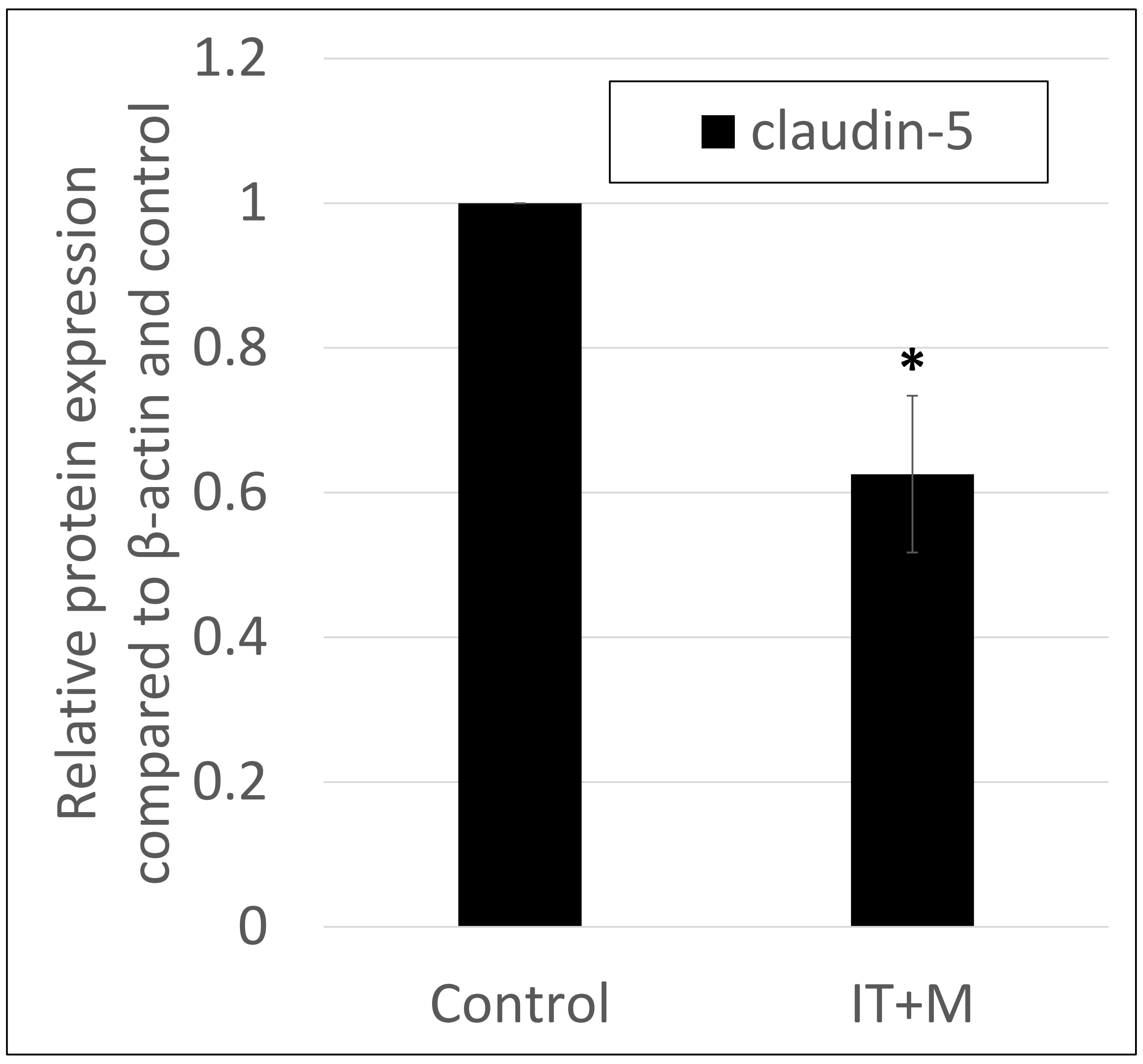


**Supplementary figure S4.** IFN-γ + TNF-α and MDP induce active IL-1β secretion and TJ disruption in CECs.
(**A**) Uncropped blots of **Figure 1D**.
(**B**) Quantification of claudin-5 expression (average +/- SEM, N = 3, * $p < 0.05$, as assessed by Student's t-test).

# Supplementary figure S5

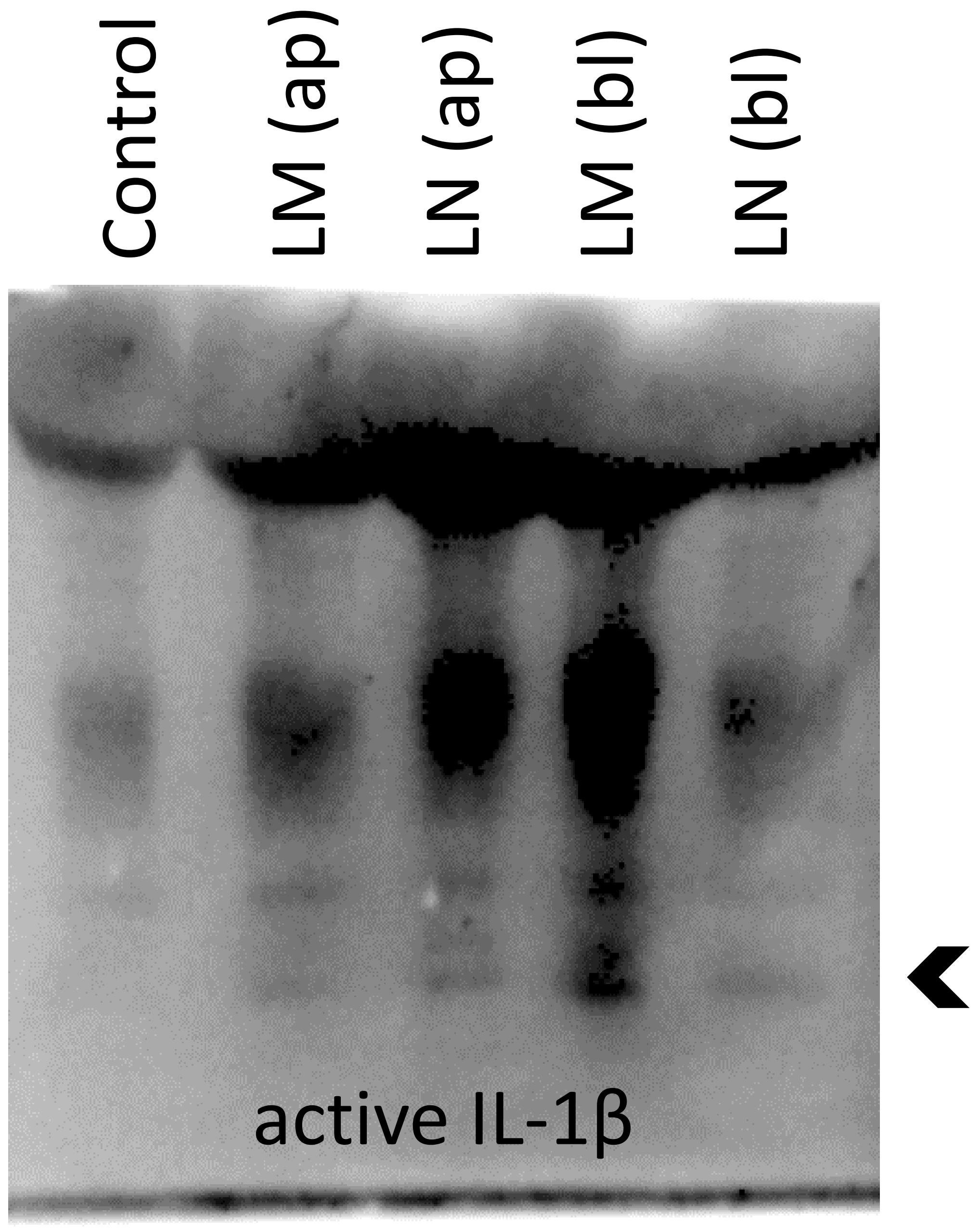


**Supplementary figure S5.** CECs respond to both apical and basolateral inflammatory stimuli.
Uncropped blots of **Figure 2A**.

## Supplementary figure S6

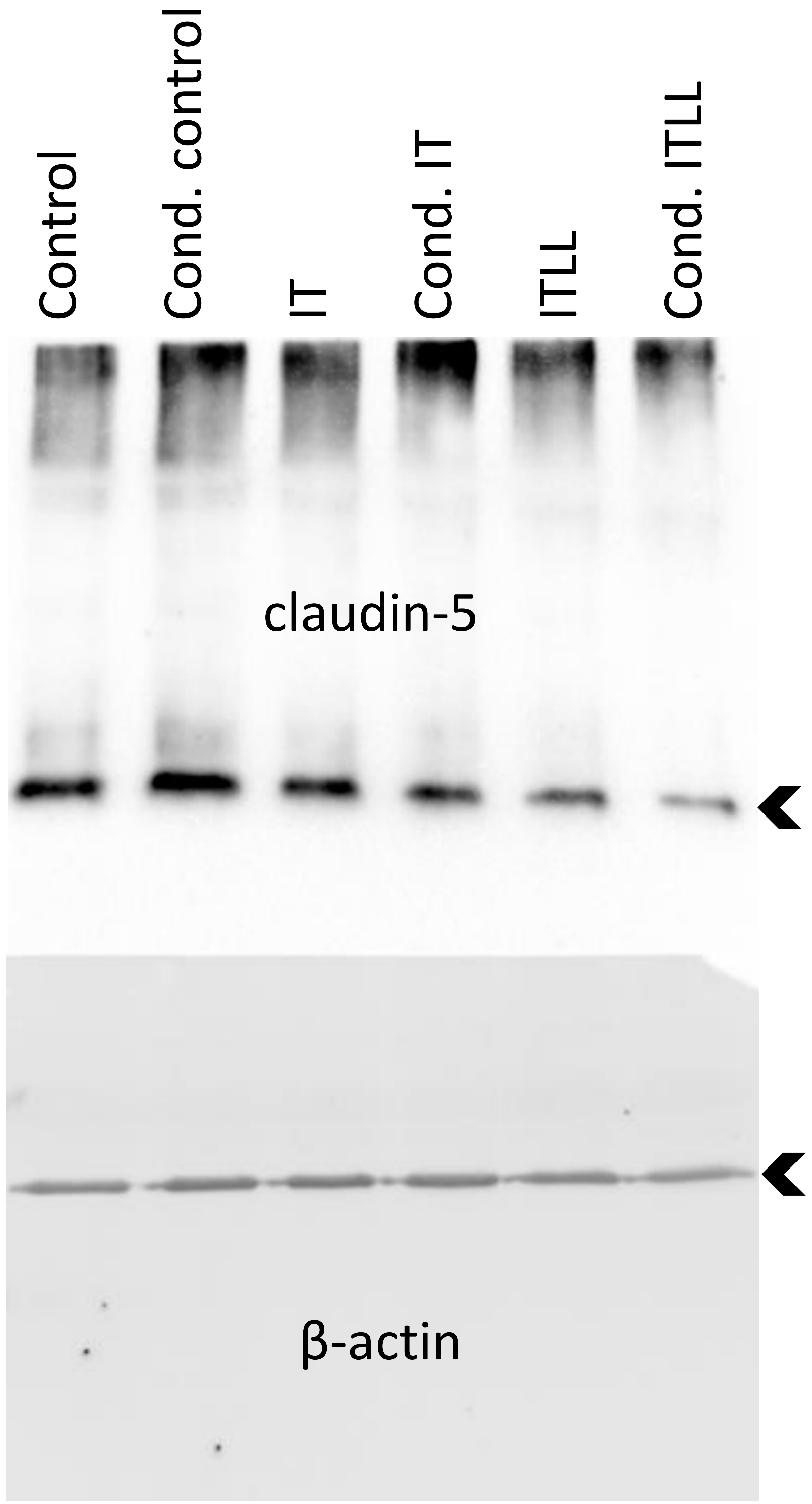


**Supplementary figure S6.** Inflammatory stimuli released by pericytes alter barrier properties of PBECs.
Uncropped blots of **Figure 3A**.

## Supplementary figure S7

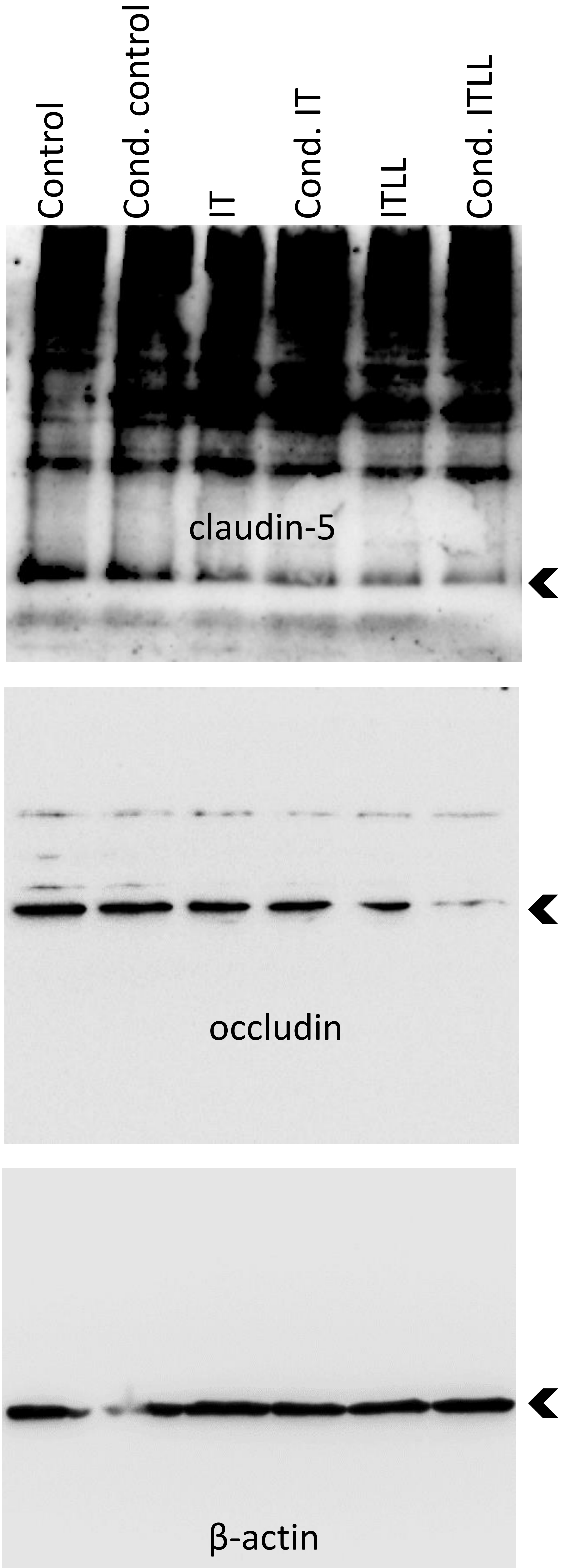


**Supplementary figure S7**. Inflammatory stimuli released by pericytes alter barrier properties of D3 cells.
Uncropped blots of **Figure 3B**.

# Supplementary figure S8

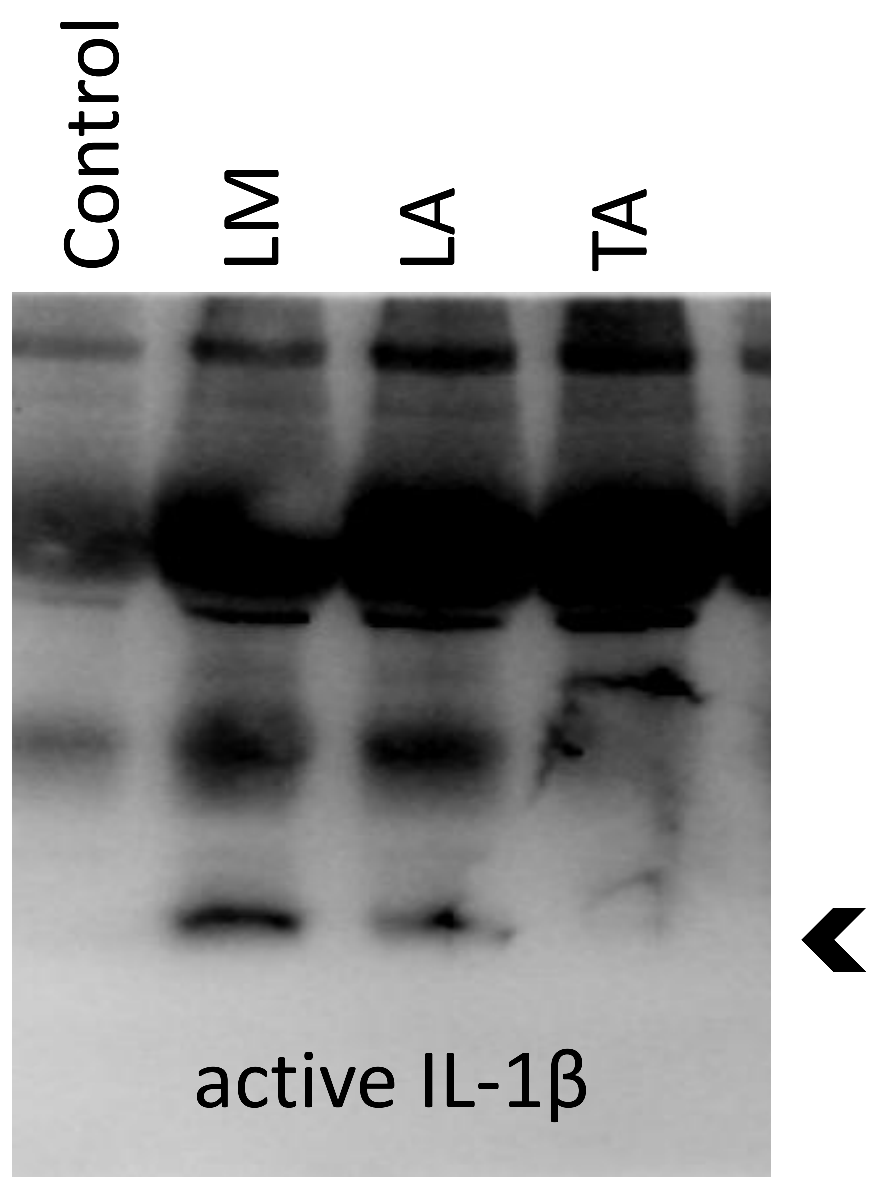


**Supplementary figure S8. Activated CECs secrete inflammatory mediators into the apical direction.**

Uncropped blots of **Figure 5A**.

## Supplementary figure S9

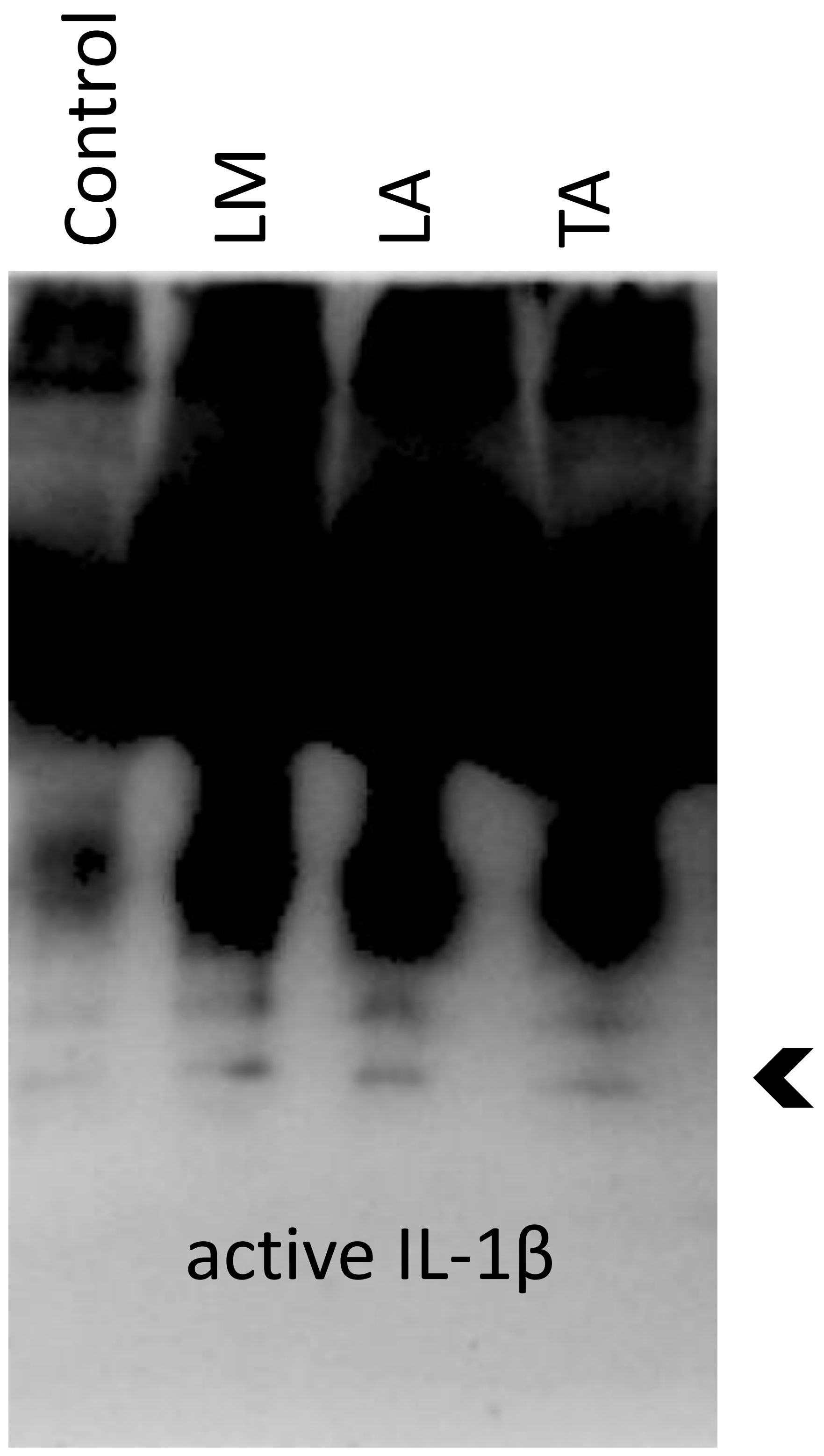


**Supplementary figure S9.** Activated CECs secrete inflammatory mediators into the basolateral direction.
Uncropped blots of **Figure 5B**.
